# Efficacy and Safety of Very Early Mobilization in Patients with Acute Stroke: A Systematic Review and Meta-analysis

**DOI:** 10.1038/s41598-017-06871-z

**Published:** 2017-07-26

**Authors:** Tao Xu, Xinyuan Yu, Shu Ou, Xi Liu, Jinxian Yuan, Yangmei Chen

**Affiliations:** Department of Neurology, the Second Affiliated Hospital of Chongqing Medical University, 76 Linjiang Road, Yuzhong District, Chongqing, 400010 China

## Abstract

Whether very early mobilization (VEM) improves outcomes in stroke patients and reduces immobilization-related complications (IRCs) is currently unknown. The objective of this systematic review and meta-analysis was to evaluate the efficacy and safety of VEM in acute stroke patients following admission. Medline, Embase, and Cochrane Central Register of Controlled Trials databases were searched for randomized controlled trials (RCTs) that examined the efficacy or safety of VEM in patients with acute stroke. VEM was defined as out of bed activity commencing within 24 or 48 hours after the onset of stroke. A total of 9 RCTs with 2,803 participants were included. Upon analysis, VEM was not associated with favorable functional outcomes (modified Ranking Scale: 0–2) at 3 months [relative risk (RR): 0.96; 95% confidence interval (CI): 0.86–1.06]; VEM did not reduce the risk of IRCs during follow up. With respect to safety outcomes, VEM was not associated with a higher risk of death (RR: 1.04; 95% CI: 0.52–2.09) and did not increase the risk of neurological deterioration or incidence of falls with injury. In conclusion, pooled data from RCTs concluded that VEM is not associated with beneficial effects when carried out in patients 24 or 48 hours after the onset of a stroke.

## Introduction

Stroke has been well established as a leading cause of mortality and disability worldwide^1^. Treatment within dedicated stroke units has significantly improved outcomes in patients with acute strokes, primarily due to reductions in mortality, disability, complications, and the need for long-term care^2, 3^. Very early mobilization (VEM) after stroke onset is thought to be an important component of stroke unit treatment, which potentially contributes to improved outcomes after acute stroke^4^. VEM is usually defined as intensive out of bed (OOB) activity comprising of sitting, standing, and walking at the earliest possible time no later than 1 or 2 days after onset^5–7^.

Due to an overall decline in brain plasticity over the course of symptom onset, the optimal period for neuronal repair may be within a narrow window after stroke onset^8^. Previous studies have reported that immobilization in bed after stroke onset may result in serious complications, such as pneumonia and deep vein thrombosis^9, 10^. Moreover, extended rest in bed has been associated with musculoskeletal issues resulting in a severe reduction in muscle mass^11^ and dysfunctions of the cardio-respiratory^12^ and immune system^13^. These negative effects caused by immobilization may slow recovery and increase mortality and morbidity in patients suffering from a stroke^9, 10, 14^. VEM therefore may improve outcomes in patients and reduce immobilization-related complications (IRCs)^7^. However, there is also concern that changes in cerebral blood flow and increased blood pressure caused by VEM may worsen stroke outcomes, as well as the frequency of falls during VEM^15, 16^.

Recent published studies have shown inconsistent results regarding the efficacy and safety of VEM after acute stroke. The most recent systematic review regarding the effects of VEM on clinical outcomes in stroke patients was conducted in 2009 and included only one randomized controlled trial (RCT)^17^. The optimal time for mobilization remains unknown, and whether VEM could improve outcomes of stroke and reduce IRCs remains unclear. Therefore, we conducted a comprehensive and updated meta-analysis to evaluate the efficacy and safety of VEM after admission for acute stroke.

## Results

### Study Selection and Characteristics

**Figure 1:**
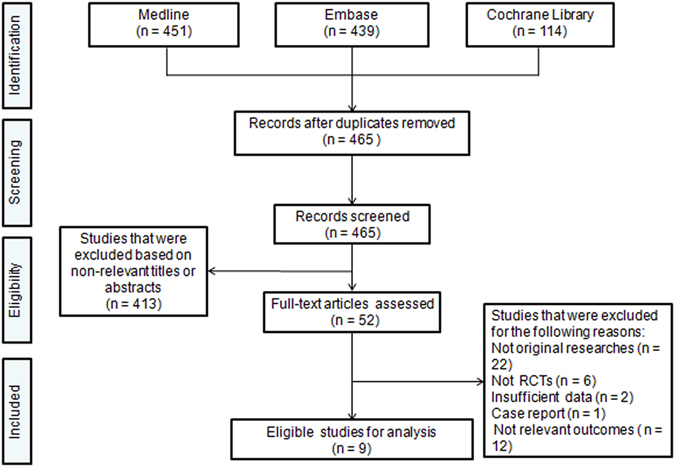
Flowchart of the literature search performed.

Of the 465 unduplicated records that were identified during our initial search, the full texts of 52 articles were reviewed. A total of 9 articles^5, 6, 15, 16, 18–22^ met our inclusion criteria and were finally included in this meta-analysis (Fig. 1). A total of 9 RCTs published between 2008 and 2016 including 2,803 participants were examined. Of the 9 included studies, 3^16, 18, 21^ were conducted in Europe, 2^19, 20^ in Asia, 1^5^ in South America, 1^6^ in Oceania, 1^22^ in 2 countries across Europe and Oceania, and 1^15^ in 5 countries across multiple continents. With respect to stroke subtype, 5^6, 15, 16, 19, 22^ studies included patients with any stroke type, 3^5, 18, 21^ included patients with ischemic stroke (IS) only, and only 1^20^ included patients with hemorrhagic stroke (HS). For definitions of VEM, 6 studies^6, 15, 16, 18, 19, 22^ defined VEM as OOB activity starting within 24 hours of stroke onset, which was compared with late mobilization (LM) starting after 24 hours of stroke onset; 2 studies^5, 20^ defined VEM as OOB activity starting within 48 hours of stroke onset, which was compared with LM starting 48 hours or 7 days after stroke onset. We also included a study^21^ that defined VEM as OOB activity starting at 52 hours of stroke onset because VEM starting at 52 hours after stroke onset was very close to that starting within 48 hours of stroke onset^7^. The detailed characteristics of the included studies are listed in Table 1.

**Table 1:** Characteristics of the included studies.

First author/Publication year	Region	Stroke subtype	Study design	Characteristics of populations and interventions		Main outcomes
				Very early mobilization	Late mobilization	
Herisson 2016^18^	France	Ischemic stroke	RCT	Age (mean): 68.1 y; SS (n): 63; MT: begin within 24 h of stroke onset; OOB activity: sitting	Age (mean): 71.2 y; SS (n): 75; MT: begin after 24 h of stroke onset; OOB activity: sitting	mRS, mortality, NIHSS, Barthel Index, and medical complications at 3 months, length of hospital stay
Chippala 2016^19^	India	Any stroke	RCT	Age (mean): 59.3 y; SS (n): 40; MT: begin within 24 h of stroke onset; OOB activity: sitting, standing, and walking	Age (mean): 60.6 y; SS (n): 40; MT: begin after 24 h of stroke onset; OOB activity: sitting, standing, and walking	Barthel Index at 3 months, length of hospital stay
Poletto 2015^5^	Brazil	Ischemic stroke	RCT	Age (mean): 64.0 y; SS (n): 18; MT: begin within 48 h of stroke onset; OOB activity: sitting, standing, and walking	Age (mean): 66.0 y; SS(n): 19; MT: begin after 48 h of stroke onset; OOB activity: sitting, standing, and walking	mRS, Barthel Index at 3 months, length of hospital stay
Bernhardt 2015 (AVERT III)^15^	Australia, New Zealand, Malaysia, Singapore, and UK	Any stroke	RCT	Age (median): 72.3 y; SS (n): 1,054; MT: begin within 24 h of stroke onset; OOB activity: sitting, standing, and walking	Age (median): 72.7 y; SS (n): 1,050; MT: begin after 24 h of stroke onset; OOB activity: sitting, standing, and walking	mRS, mortality, and medical complications at 3 months, length of hospital stay
Liu 2014^20^	China	Hemorrhagic stroke	RCT	Age (mean): 58.5 y; SS (n): 122; MT: begin within 48 h of stroke onset; OOB activity: exercises and functional training	Age (mean): 59.1 y; SS (n): 121; MT: begin after 7 d of stroke onset; OOB activity: exercises and functional training	Mortality and Barthel Index at 3 months, length of hospital stay
Sundseth 2012^16^	Norway	Any stroke	RCT	Age (mean): 76.5 y; SS (n): 27; MT: begin within 24 h of stroke onset; OOB activity: NA	Age (mean): 77.3 y; SS (n): 29; MT: begin after 24 h of stroke onset; OOB activity: NA	mRS, mortality, Barthel Index, NIHSS, and medical complications at 3 months
Diserens 2012^21^	Switzerland	Ischemic stroke	RCT	Age (mean): 72.0 y; SS (n): 25; MT: begin at 52 h of stroke onset; OOB activity: sitting, standing, and walking	Age (mean): 71.0 y; SS (n): 17; MT: begin after 7 d of stroke onset; OOB activity: sitting, standing, and walking	mRS, mortality, and medical complications at 3 months, length of hospital stay
Langhorne 2010 (VERITAS)^22^	Australia and UK	Any stroke	RCT	Age (median): 64.0 y; SS (n): 16; MT: begin within 24 h of stroke onset; OOB activity: sitting, standing, and walking	Age (median): 71.0 y; SS (n): 16; MT: begin after 24 h of stroke onset; OOB activity: sitting, standing, and walking	mRS, mortality, Barthel Index, and medical complications at 3 months, length of hospital stay
Bernhardt 2008 (AVERT II)^6^	Australia	Any stroke	RCT	Age (median): 74.6 y; SS (n): 38; MT: begin within 24 h of stroke onset; OOB activity: sitting, standing, and walking	Age (median): 74.9 y; SS (n): 33; MT: begin after 24 h of stroke onset; OOB activity: sitting, standing, and walking	mRS, mortality, and medical complications at 3 months, length of hospital stay

Abbreviations: RCT, randomized controlled trial; SS, sample size; MT, mobilization time; OOB, out of bed; NA, not available; mRS, modified Rankin Scale.

### Efficacy and Safety Outcomes

**Figure 2:**
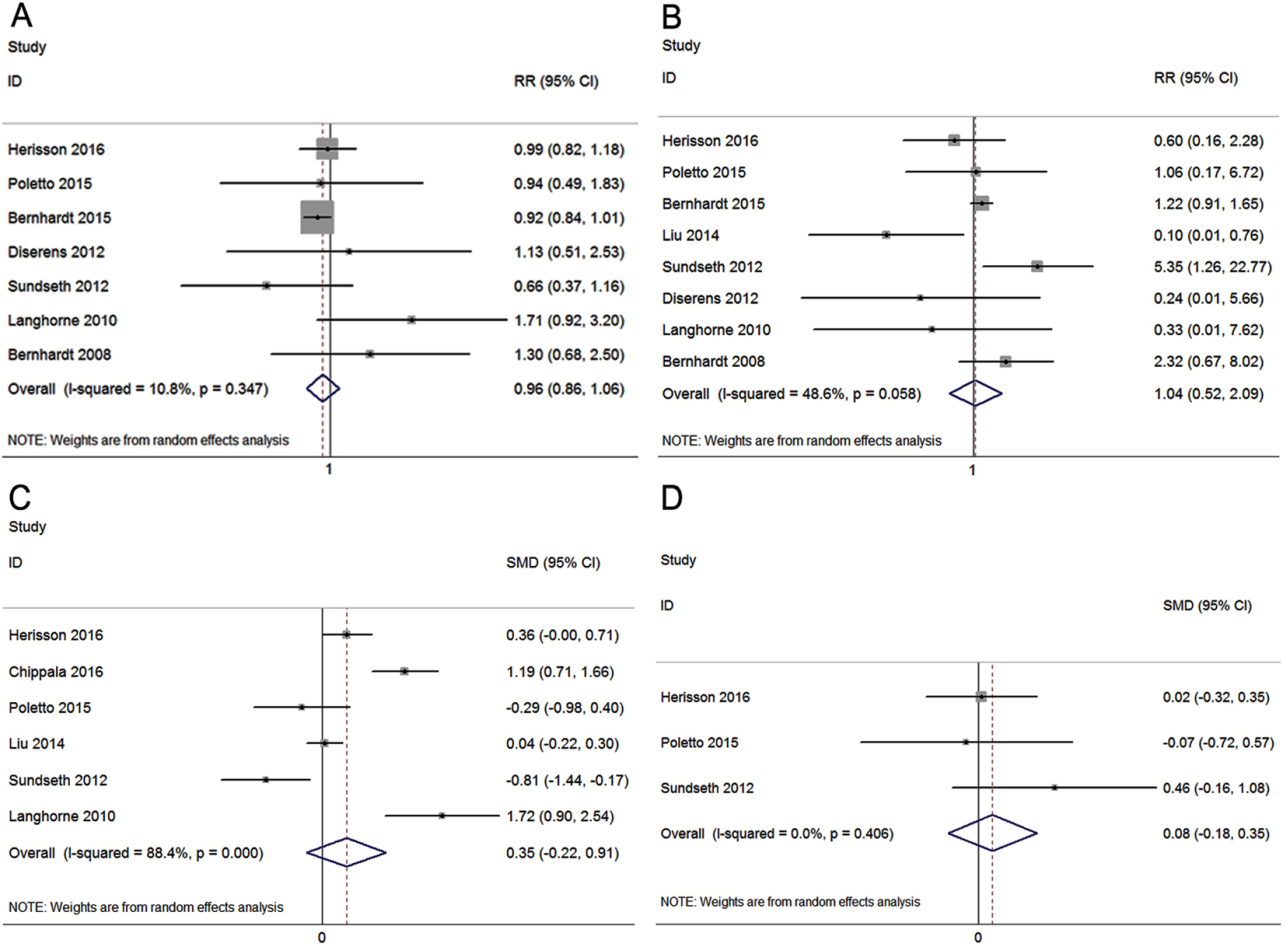
Forest plots of efficacy and safety outcomes of very early mobilization at 3 months. The diamond indicates the estimated relative risk (RR) or standardized mean differences (SMD) (95% confidence interval). The p-value showed on each figure is for heterogeneity test. The modified Rankin Scale (0–2): p for overall effect = 0.40, Q = 6.73 **(A)**. Mortality: p for overall effect = 0.90, Q = 13.62 **(B)**. Barthel Index: p for overall effect = 0.23, Q = 43.22 **(C)**. National Institutes of Health Stroke Scale: p for overall effect = 0.54, Q = 0.11 **(D)**.

Figure 2A demonstrated that VEM was not associated with a significant change in the primary efficacy outcome [modified Rankin Scale (mRS): 0–2] at 3 months [relative risk (RR): 0.96; 95% confidence interval (CI): 0.86–1.06]. For the primary safety outcomes (mortality at 3 months), VEM was not associated with a significantly higher risk of death (RR: 1.04; 95% CI: 0.52–2.09; Fig. 2B).

No significant differences were observed in any secondary efficacy outcome measurements, including Barthel Index scores at 3 months [standardized mean differences (SMD): 0.35; 95% CI: −0.22–0.91; Fig. 2C], National Institutes of Health Stroke Scale (NIHSS) scores at 3 months (SMD: 0.08; 95% CI: −0.18–0.35; Fig. 2D), or IRCs during follow up (detailed estimates listed in Table 2) between the VEM and LM groups. The length of hospital stay in the VEM group was shorter than that in the LM group (SMD: −0.58; 95% CI: −0.96–−0.19; Table 2). For secondary safety outcomes, VEM was not associated with a substantially higher risk of neurological deterioration (RR: 0.79; 95% CI: 0.39–1.60; Table 2) or falls with injury (RR: 0.91; 95% CI: 0.53–1.55; Table 2).

**Table 2:** Secondary efficacy and safety outcomes.

Groups	No. of studies	Estimates	95% CI	p-value for OE	*I* ^2^, %	p-value for heterogeneity	Q-value
**Secondary efficacy outcomes**		SMD					
Length of hospital stay	8	−0.58	−0.96–−0.19	<0.01	89.7	<0.01	68.09
IRCs		RR					
Pulmonary infection	5	0.81	0.40–1.64	0.56	0.00	0.50	3.34
Urinary tract infection	3	0.82	0.11–5.90	0.85	50.3	0.13	4.02
Deep vein thrombosis	1	3.48	0.14–83.83	0.44	None	None	None
Pulmonary embolism	1	0.23	0.01–5.35	0.36	None	None	None
**Secondary safety outcomes**							
Neurological deterioration	4	0.79	0.39–1.60	0.52	57.9	0.07	7.13
Falls	3	0.91	0.53–1.55	0.73	5.7	0.35	2.12

Abbreviations: RR, relative risk; SMD, standardized mean difference; CI, confidence interval; OE, overall effect; IRC, immobilization-related complication.

### Subgroup, Heterogeneity, and Sensitivity Analyses

**Figure 3:**
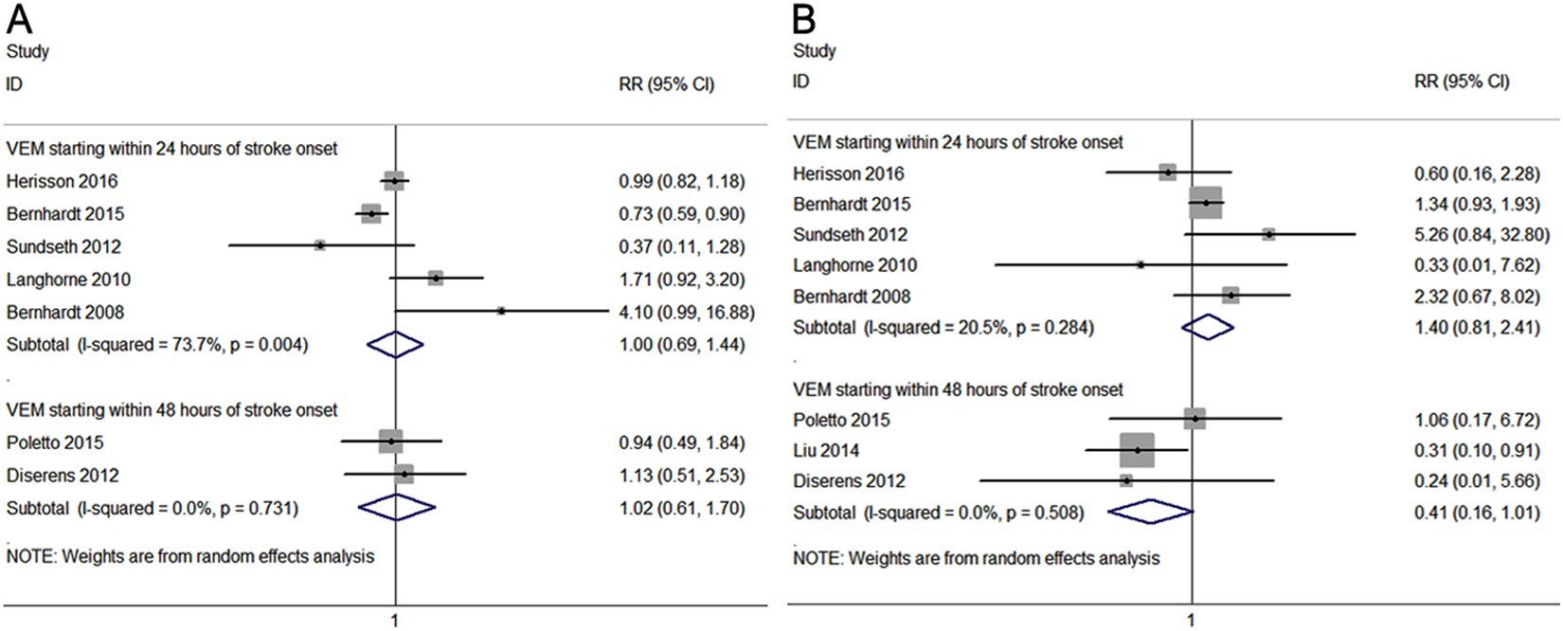
Forest plots of primary efficacy and safety outcomes stratified by starting time of very early mobilization. The diamond indicates the estimated relative risk (RR) (95% confidence interval). The p-value showed on each figure is for heterogeneity test. The modified Rankin Scale (0–2) at 3 months: within 24 hours, p for overall effect = 0.98, Q = 15.20; within 48 hours, p for overall effect = 0.95, Q = 0.12 **(A)**. Mortality stratified by starting time of VEM: within 24 hours, p for overall effect = 0.23, Q = 5.03; within 48 hours, p for overall effect = 0.05, Q = 1.35 **(B)**.

**Figure 4:**
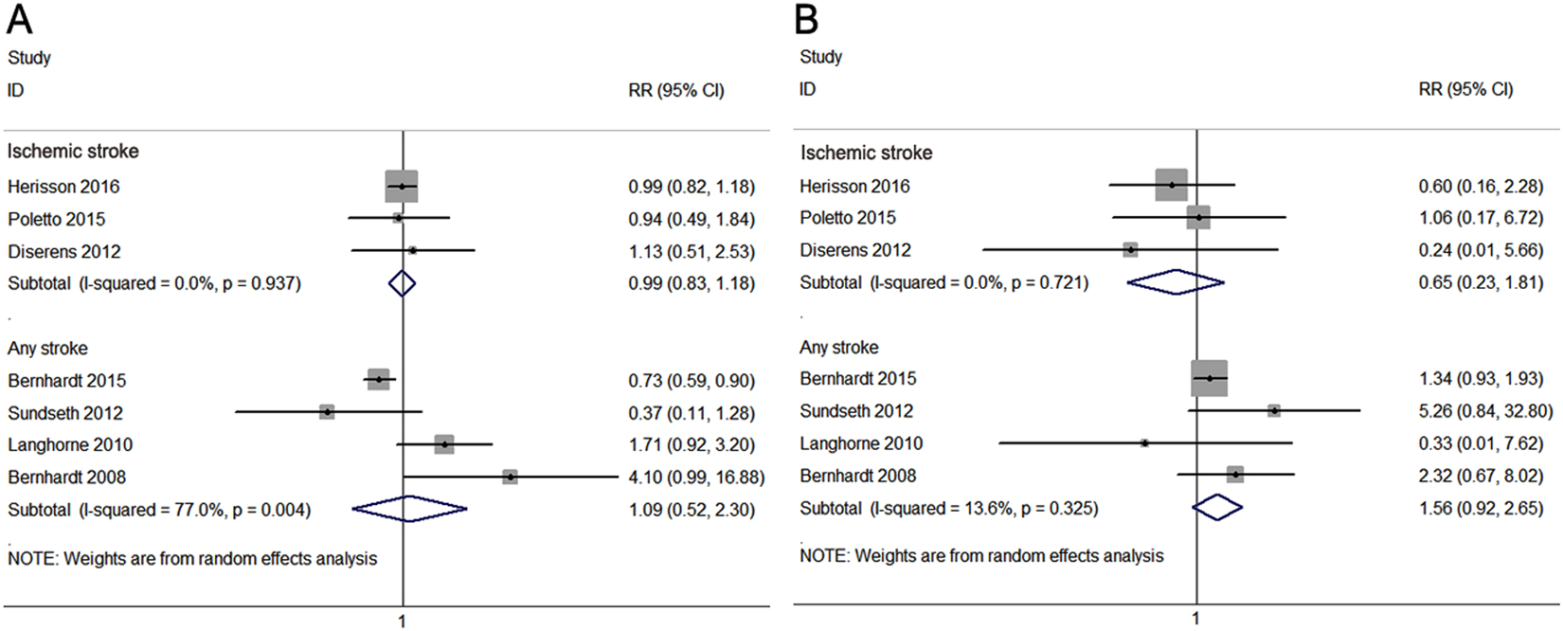
Forest plots of primary efficacy and safety outcomes stratified by stroke type. The diamond indicates the estimated relative risk (RR) (95% confidence interval). The p-value showed on each figure is for heterogeneity test. Modified Rankin Scale (0–2) at 3 months: ischemic stroke, p for overall effect = 0.90, Q = 0.13; any stroke, p for overall effect = 0.82, Q = 13.07 **(A)**. Mortality at 3 months: ischemic stroke, p for overall effect = 0.40, Q = 0.65; any stroke, p for overall effect = 0.10, Q = 3.47 **(B)**.

We conducted subgroup analyses according to time of start mobilization and stroke subtype to evaluate the potential effect modification of the two key variables on primary efficacy and safety outcomes. Neither mobilization commencing within 24 hours of stroke onset nor mobilization commencing within 48 hours of stroke onset were associated with a significant change of primary efficacy outcome (RR of VEM starting within 24 hours: 1.00, 95% CI: 0.69–1.44; RR of VEM starting within 48 hours: 1.02, 95% CI: 0.61–1.70; Fig. 3A) or mortality at 3 months (RR of VEM starting within 24 hours: 1.40, 95% CI: 0.81–2.41; RR of VEM starting within 48 hours: 0.41, 95% CI: 0.16–1.01; Fig. 3B). The subgroup analyses of stroke subtype indicated that, in the subtypes of IS and any strokes, VEM had no significant effect on primary efficacy outcomes (RR of IS: 0.99, 95% CI: 0.83–1.18; RR of any stroke: 1.09, 95% CI: 0.52–2.30; Fig. 4A) or mortality at 3 months (RR of IS: 0.65, 95% CI: 0.23–1.81; RR of any stroke: 1.56, 95% CI: 0.92–2.65; Fig. 4B). There were insufficient data to conduct a subgroup analysis for the effect of VEM on HS outcome because only one study^20^ investigated the efficacy and safety of VEM in patients with HS. Moreover, the quality of the included studies varied, and the study by Herisson *et al*. had a relatively high risk of bias. When we excluded the study by Herisson *et al*., the pooled results of the meta-analysis showed no significant changes. The omission of any single study did not significantly alter pooled RRs or heterogeneity.

### Publication Bias

Visual inspection of the funnel plots identified asymmetry (see Supplementary Figs S1 and S3). Further Begg’s and Egger’s tests showed no significant evidence of publication bias in our meta-analysis (see Supplementary Figs S2 and S4).

### Risk of Bias

**Figure 5:**
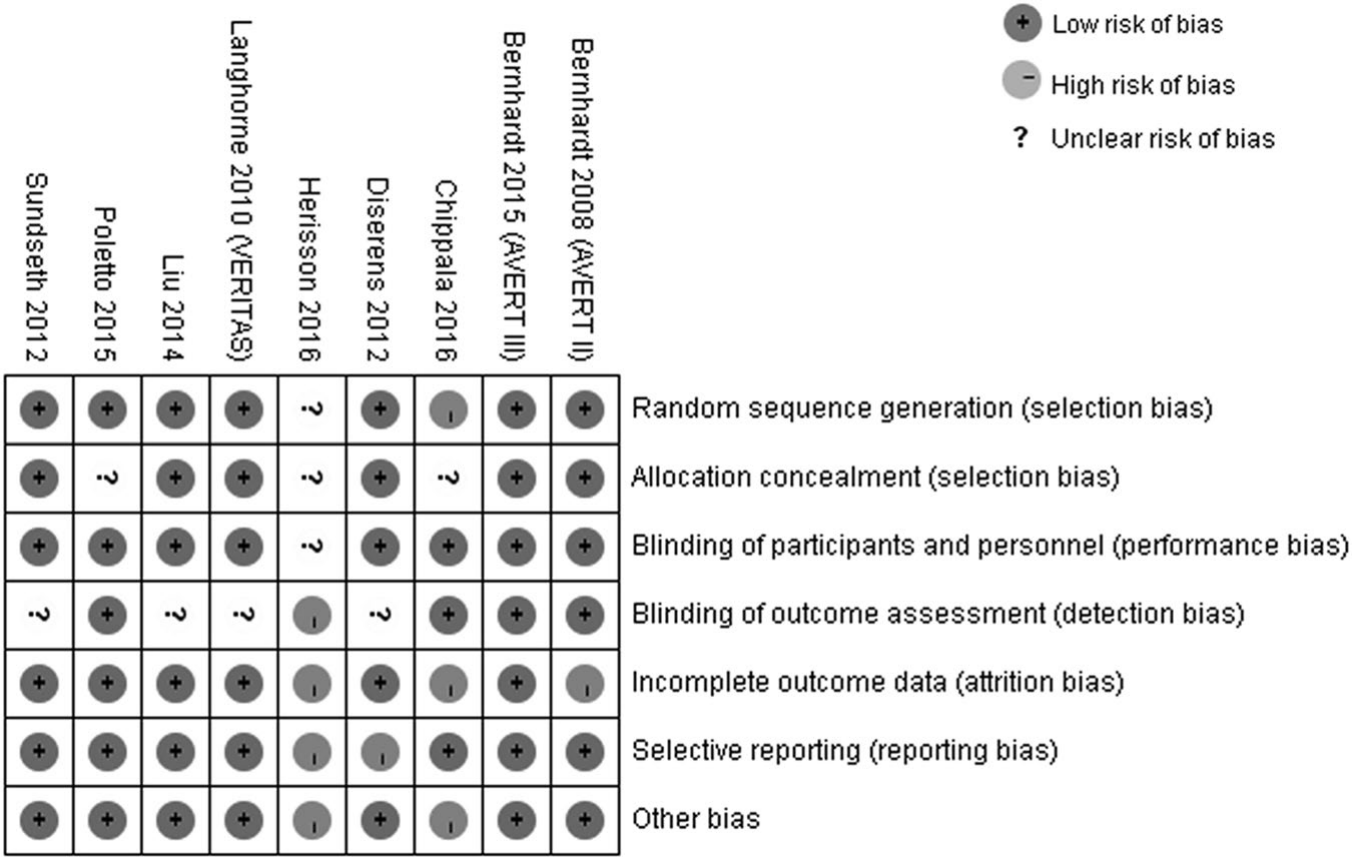
Risk of bias: A summary table for each risk of bias item for each study.

Details regarding the risks of bias of the included studies are shown in Fig. 5. Of the eligible studies, 8 described random sequence generation (low risk = 7^5, 6, 15, 16, 20–22^, high risk = 1^19^). Six studies^6, 15, 16, 20–22^ had a low risk of bias in allocation concealment, and 8 trials^5, 6, 15, 16, 19–22^ had a low risk of bias in blinding participants and researchers. Five trials described the risk of bias in the blinding of outcome assessment (low risk = 4^5, 6, 15, 19^, high risk = 1^18^). Six trials^5, 15, 16, 20–22^ had a low risk of bias for incomplete outcome data. For selective reporting domains, seven studies^5, 6, 15, 16, 19, 20, 22^ were judged as having a low risk of bias (Fig. 5).

## Discussion

From the publication of the first RCT examining VEM in stroke patients in 2008 (AVERT, Phase II)^6^, the correlation between VEM and improved outcomes, as well as earlier short-term functional recovery, has been of clinical interest. VEM in stroke units is recommended in a range of European, American, and Asian guidelines as a strategy to minimize or prevent IRCs^7^. However, the adoption and implementation of VEM after stroke has also generated controversy worldwide because of the limited evidence base to support VEM in acute stroke patients. A total of 2,803 patients from 9 RCTs were included in the present systematic review and meta-analysis. Our meta-analysis findings indicate that the efficacy of VEM on outcomes in stroke patients may be questionable. Although a pooled estimation in this meta-analysis indicated that VEM could shorten the length of hospital stay, VEM had no significant benefit in any of the functional recovery outcomes examined, including mRS (0–2), NIHSS, and Barthel Index scores at 3 months follow-up. Additionally, VEM did not lower the risk of IRCs. The length of hospital stay is an indirect outcome regarding stroke recovery, which is easily affected by patient motivation and clinical decisions. Therefore, only a shorter duration of hospital stay in the VEM group cannot support the efficacy of VEM in patients with stroke. With respect to safety outcomes, VEM was not associated with a higher risk of death at 3 months and neurological deterioration or falls with injury during follow up.

The majority of studies involving VEM aimed to determine the optimal time to commence mobilization in patients following stroke. Animal models of stroke have indicated an early post-stroke phase of increased brain plasticity, suggesting that this may be a crucial time for intervention^8, 23–25^. The time window for therapeutic intervention and repair may be narrow due to a short period of neuronal plasticity after stroke^6^. Therefore, we assumed that prompt commencement of OOB activity may improve functional outcomes after stroke onset. However, Krakauer and colleagues^8^ assumed that the forced use of the paralytic limbs prematurely (within 1–3 days) after brain injury may block potentially beneficial plasticity changes because too early mobilization may weaken GABA-mediated tonic inhibition. Reducing GABA-mediated inhibition in the first few days after stroke onset may influence the focus size of the infarct^8^. Based on the current understanding of brain plasticity and repair^7, 15^, the optimal time to commence rehabilitation remains unclear. With respect to the clinical practice of VEM, Bernhardt and colleagues found that most acute stroke guidelines have not defined VEM criteria due to the uncertainty of the optimal time to initiate rehabilitation following stroke onset; only a few recent published stroke guidelines recommended commencement of mobilization within 24 hours or 48 hours of stroke onset^7^. However, according to the results from the presented meta-analysis, both of these recommendations have insufficient evidence.

Early OOB activity was previously thought to decrease the risk of IRCs in patients with stroke. However, no RCT to date (as well as our presented meta-analysis) have demonstrated that VEM has a significant effect on IRCs. One interpretation for this finding is that the primary factor influencing the risk of IRCs may not be the time point at which mobilization is initiated but other factors, such as age, stroke severity, and the dose and pattern of mobilization that are associated with the risk of IRCs.

Based on subgroup analyses of stroke subtypes, VEM could not improve outcomes in patients with IS. Only one RCT^20^ with a small sample size investigated the effect of VEM on outcomes in HS patients. There was insufficient data for us to conduct a subgroup analysis regarding outcomes in HS patients. Therefore, whether early OOB activity can significantly improve the outcomes of HS patients is uncertain. Additionally, 56% (n = 5) of included RCTs^6, 15, 16, 19, 22^ did not report clinical details regarding stroke subtype, which may reduce the strength of the results and the conclusions drawn from them. A clear stroke subtype classification should be mandatory in future RCTs.

Due to the complexity and uncertainty of the degree of neuroplasticity following stroke, the current definition of VEM for patients with stroke is, arguably, too simplistic. The presented meta-analysis and the included RCTs compared the efficacy and safety of the VEM group with a usual care group after stroke onset and discussed whether commencement of mobilization within 24 or 48 hours was associated with favorable stroke outcomes. However, these studies did not explore a dose-response relationship between commencement time of mobilization and magnitude of functional recovery in patients with stroke. Moreover, the commencement time in conjunction with the dose (e.g., daily frequency and lasting time) of mobilization may be important for stroke recovery^8, 24^. In the present meta-analysis, 89% (n = 8) of included RCTs^5, 6, 16, 18–22^ discussed the start time of VEM but did not analyze how to control the dose and pattern of mobilization in their studies, which may reduce the strength of their results. A recent non-RCT study with a dose-response analysis indicated that shorter and more frequent mobilization within 24 hours of acute stroke was associated with improved favorable outcomes at 3 months; notably, when keeping the time to VEM (stating within 24 hours) and daily amount (total lasting time of mobilization) constant, increasing the frequency of VEM could improve favorable function outcome ^26^. The appropriate pattern of VEM should also be emphasized for stroke recovery and should include active sitting, standing, and walking activity, with a titrating dose [e.g., from low arousal (active sitting) to high function (standing and walking); from low frequency to high frequency]^24, 26^. These studies suggest that future VEM research and trials should be conducted with a dose-response design.

Our meta-analysis has several limitations that should be considered when interpreting the results. Firstly, in addition to the RCT conducted by Bernhardt and colleagues (AVERT, Phase III, 2015)^15^, other included studies have relatively small sample sizes, which may influence the reliability of the results. Due to this bias, both a random-effects model to pool the estimates and sensitivity analysis were performed to reduce its effect on the results of the meta-analysis. Sensitivity analysis indicates that even after excluding the RCT by Bernhardt and colleagues (2015), the pooled results of the meta-analysis had no significant changes. Secondly, only nine studies are included in this meta-analysis, which may reduce the strength of our results. Thirdly, the primary and secondary endpoints from the included RCTs were primarily assessed at 3 months after stroke onset, which did not enable us to analyze dynamic changes regarding the efficacy and safety of VEM in stroke patients. Fourthly, not all included studies evaluated efficacy and safety based on sex; thus, we could not evaluate gender differences. Moreover, all included studies included patients over 18 years of age with stroke, which did not enable us to analyze the efficacy and safety of VEM in children and teens with stroke.

In summary, VEM did not improve functional outcomes or reduce the risk of IRCs. VEM was also not associated with higher risks of mortality, stroke progression or falls with injury. Therefore, according to our meta-analysis, convincing evidence to support VEM in patients with acute stroke is lacking. VEM is therefore not recommended as a component of stroke-unit treatment. Thus, well-designed RCTs with dose-response analysis are warranted to evaluate the efficacy and safety of early OOB activity in patients with acute stroke. Important factors that may affect the prognosis of stroke should be considered when a RCT is designed, such as the dose and pattern of mobilization, stroke subtypes, and age.

## Methods

A systematic review and meta-analysis were conducted according to the previously published guidelines for the preferred reporting items for systematic reviews and meta-analyses (PRISMA)^27^.

### Search Strategy

The Medline, Embase, and Cochrane Central Register of Controlled Trials (CENTRAL) electronic databases were searched using predefined terms and search criteria. The latest search was conducted on October 8, 2016. The following search terms used were in the Medline database: (mobilization [All Fields] OR rehabilitation [Mesh Terms]) AND (early [All Fields]) AND (stroke [Mesh Terms] OR cerebrovascular disorders [Mesh Terms]). The search strategy for the Embase and CENTRAL database was similar to that used for the Medline database. Additional relevant articles were obtained by searching the reference lists of the articles included in this study. No language or publication status restrictions were imposed.

### Study selection, Data Extraction, and Quality Assessment

This section was conducted by 2 of the authors (T.X., and X.Y.Y.) independently. The inclusion criteria for the meta-analysis were: (1) RCT design; (2) evaluating the efficacy or safety of VEM compared with LM in patients with acute stroke; (3) presented the data necessary for calculating RRs or SMDs, and 95% confidence intervals (CIs). We included studies that defined VEM as OOB activity starting within 24 or 48 hours after stroke onset^7^. Non-original articles, articles with insufficient data or irrelevant outcomes, and case reports were excluded. The following data were extracted from each study independently by us: first author, publication year, regions, subtype of stroke, study design, population demographics, characteristics of interventions and comparisons, and outcomes of stroke.

We used the uniform criteria of the Cochrane collaboration to assess the risk of bias in RCTs^28^. The evaluative criteria included seven domains, which are listed in Fig. 5. For each domain, we judged the quality of each RCT as high or low or unclear risk of bias. Review Manager 5.3 software (Cochrane Collaboration, UK) was used for quality assessment.

### Outcomes Definition

The primary efficacy outcome was the favorable outcome of functional recovery, which was defined as a mRS score of 0–2 at 3 months. The secondary efficacy outcomes were the length of hospital stay, the scores of NIHSS and the Barthel Index at 3 months, and IRCs during follow-up (pulmonary infection, urinary tract infection, deep vein thrombosis, and pulmonary embolism). The primary safety outcome was mortality at 3 months. The secondary safety outcomes were neurological deterioration and falls with injury. Neurological deterioration included recurrent stroke and stroke progression, which was assessed according to new neurological deficits and NIHSS during follow up.

### Statistical Analysis

STATA version 12.0 (StataCorp, College Station, TX, USA) was used for the statistical analyses. For dichotomous outcomes of mRS (0–2) and morality at 3 months, IRCs, falls, and neurological deterioration, RR with 95% CI was calculated as the overall effect measure. For continuous outcomes of NIHSS scores, Barthel Index scores and the length of hospital stay and SMD with 95% CI [the input data was the mean, standard deviation (SD), and sample size (n)] was used as the overall effect measure. When the mean and standard deviation (e.g., continuous data was presented as the median and range) were not reported, we used the methodology described by Hozo *et al*.^29^ to calculate the mean and SD. We recognize the potential diversities in demographics of patients (e.g., age, sex, ethnicity, territory, and sample size) between the included studies; thus, we used a random-effects model to pool the estimates^30^. The *I* ^2^ heterogeneity test was conducted to determine the magnitude of statistical heterogeneity between estimates as ([Q-degrees of freedom]/Q) × 100%, with Q representing the χ^2^ distribution^31^. To control the quality of the results in this meta-analysis, a sensitivity analysis was conducted according to the methods recommended for Cochrane systematic reviews. Each pooled analysis of efficacy and safety outcomes was reanalyzed with the exclusion of each individual study to determine the effect of a single study on the pooled estimation. In addition, to examine the effect of study quality on the outcome of the meta-analysis, the risks of bias of the included studies were also used as a basis of exclusion in sensitivity analysis. Moreover, subgroup analyses were performed to identify the effects of subgroups regarding the main variables (e.g., starting time of VEM and stroke type) on the quality of results, which was achieved by comparing the results of the meta-analysis after the exclusion of each subgroup. A p-value of less than 0.05 was considered statistically significant. Publication bias was investigated visually with funnel plots and statistically with Begg’s^32^ and Egger’s^33^ tests.

## Electronic supplementary material

Supplemental Information

## References

[1] O’Donnell MJ (2016). Global and regional effects of potentially modifiable risk factors associated with acute stroke in 32 countries (INTERSTROKE): a case-control study. Lancet (London, England).

[2] Langhorne P (2013). Stroke unit care benefits patients with intracerebral hemorrhage: systematic review and meta-analysis. Stroke; a journal of cerebral circulation.

[3] Langhorne P, de Villiers L, Pandian JD (2012). Applicability of stroke-unit care to low-income and middle-income countries. The Lancet. Neurology.

[4] van Wijk R, Cumming T, Churilov L, Donnan G, Bernhardt J (2012). An early mobilization protocol successfully delivers more and earlier therapy to acute stroke patients: further results from phase II of AVERT. Neurorehabilitation and neural repair.

[5] Poletto SR (2015). Early mobilization in ischemic stroke: a pilot randomized trial of safety and feasibility in a public hospital in Brazil. Cerebrovascular diseases extra.

[6] Bernhardt J, Dewey H, Thrift A, Collier J, Donnan G (2008). A very early rehabilitation trial for stroke (AVERT): phase II safety and feasibility. Stroke; a journal of cerebral circulation.

[7] Bernhardt J, English C, Johnson L, Cumming TB (2015). Early mobilization after stroke: early adoption but limited evidence. Stroke; a journal of cerebral circulation.

[8] Krakauer JW, Carmichael ST, Corbett D, Wittenberg GF (2012). Getting neurorehabilitation right: what can be learned from animal models?. Neurorehabilitation and neural repair.

[9] Indredavik B, Rohweder G, Naalsund E, Lydersen S (2008). Medical complications in a comprehensive stroke unit and an early supported discharge service. Stroke; a journal of cerebral circulation.

[10] McLean DE (2004). Medical complications experienced by a cohort of stroke survivors during inpatient, tertiary-level stroke rehabilitation. Archives of physical medicine and rehabilitation.

[11] Parry SM, Puthucheary ZA (2015). The impact of extended bed rest on the musculoskeletal system in the critical care environment. Extreme physiology & medicine.

[12] Herkner, H., Arrich, J., Havel, C. & Mullner, M. Bed rest for acute uncomplicated myocardial infarction. *The Cochrane database of systematic reviews*, CD003836 (2007). 10.1002/14651858.CD003836.pub2 PMC8406913 17443530

[13] Brower RG (2009). Consequences of bed rest. Critical care medicine.

[14] Klein K, Mulkey M, Bena JF, Albert NM (2015). Clinical and psychological effects of early mobilization in patients treated in a neurologic ICU: a comparative study. Critical care medicine.

[15] Bernhardt J (2015). Efficacy and safety of very early mobilisation within 24 h of stroke onset (AVERT): a randomised controlled trial. Lancet (London, England).

[16] Sundseth A, Thommessen B, Ronning OM (2012). Outcome after mobilization within 24 hours of acute stroke: a randomized controlled trial. Stroke; a journal of cerebral circulation.

[17] Bernhardt, J., Thuy, M. N., Collier, J. M. & Legg, L. A. Very early versus delayed mobilisation after stroke. *The Cochrane database of systematic reviews*, CD006187 (2009). 10.1002/14651858.CD006187.pub2 PMC6465040 19160268

[18] Herisson F (2016). Early Sitting in Ischemic Stroke Patients (SEVEL): A Randomized Controlled Trial. PloS one.

[19] Chippala P, Sharma R (2016). Effect of very early mobilisation on functional status in patients with acute stroke: a single-blind, randomized controlled trail. Clinical rehabilitation.

[20] Liu N (2014). Randomized controlled trial of early rehabilitation after intracerebral hemorrhage stroke: difference in outcomes within 6 months of stroke. Stroke; a journal of cerebral circulation.

[21] Diserens K (2012). Early mobilization out of bed after ischaemic stroke reduces severe complications but not cerebral blood flow: a randomized controlled pilot trial. Clinical rehabilitation.

[22] Langhorne P (2010). Very early rehabilitation or intensive telemetry after stroke: a pilot randomised trial. Cerebrovascular diseases (Basel, Switzerland).

[23] Zeiler SR (2016). Paradoxical Motor Recovery From a First Stroke After Induction of a Second Stroke: Reopening a Postischemic Sensitive Period. Neurorehabilitation and neural repair.

[24] Stinear, C. M. Stroke rehabilitation research needs to be different to make a difference. *F1000Research* **5** (2016). 10.12688/f1000research.8722.1 PMC4920210 27408689

[25] Murphy TH, Corbett D (2009). Plasticity during stroke recovery: from synapse to behaviour. Nature reviews. Neuroscience.

[26] Bernhardt J (2016). Prespecified dose-response analysis for A Very Early Rehabilitation Trial (AVERT). Neurology.

[27] Moher D, Liberati A, Tetzlaff J, Altman DG (2009). Preferred reporting items for systematic reviews and meta-analyses: the PRISMA statement. BMJ (Clinical research ed.).

[28] Higgins JP (2011). The Cochrane Collaboration’s tool for assessing risk of bias in randomised trials. BMJ (Clinical research ed.).

[29] Hozo SP, Djulbegovic B, Hozo I (2005). Estimating the mean and variance from the median, range, and the size of a sample. BMC medical research methodology.

[30] Borenstein M, Higgins JP, Hedges LV, Rothstein HR (2017). Basics of meta-analysis: I2 is not an absolute measure of heterogeneity. Research synthesis methods.

[31] Higgins JP, Thompson SG, Deeks JJ, Altman DG (2003). Measuring inconsistency in meta-analyses. BMJ (Clinical research ed.).

[32] Begg CB, Mazumdar M (1994). Operating characteristics of a rank correlation test for publication bias. Biometrics.

[33] Egger M, Davey Smith G, Schneider M, Minder C (1997). Bias in meta-analysis detected by a simple, graphical test. BMJ (Clinical research ed.).

