# Supplementary material for: Efficacy and Safety of Very Early Mobilization in Patients with Acute Stroke: A Systematic Review and Meta-analysis
Source: Sci Rep. 2017 Jul 26;7:6550. doi: 10.1038/s41598-017-06871-z (PMC5529532; doi:10.1038/s41598-017-06871-z)
Supplement: Supplementary file 1 — Supplemental Information [file 41598_2017_6871_MOESM1_ESM.pdf]

Supplementary Information for Efficacy and Safety of Very Early Mobilization in Patients with Acute Stroke: A Systematic Review and Meta-analysis

Title of manuscript:

Efficacy and Safety of Very Early Mobilization in Patients with Acute Stroke: A Systematic Review and Meta-analysis

Tao Xu, Xinyuan Yu, Shu Ou, Xi Liu, Jinxian Yuan & Yangmei Chen

Department of Neurology, the Second Affiliated Hospital of Chongqing Medical University, 76 Linjiang Road, Yuzhong District, Chongqing, 400010, China.

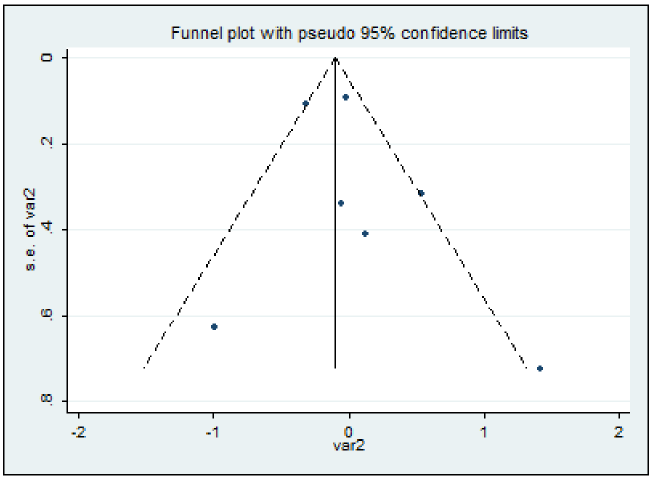

Figure S1. Funnel plot for publication bias test for mRS (0-2) at 3 months.

A

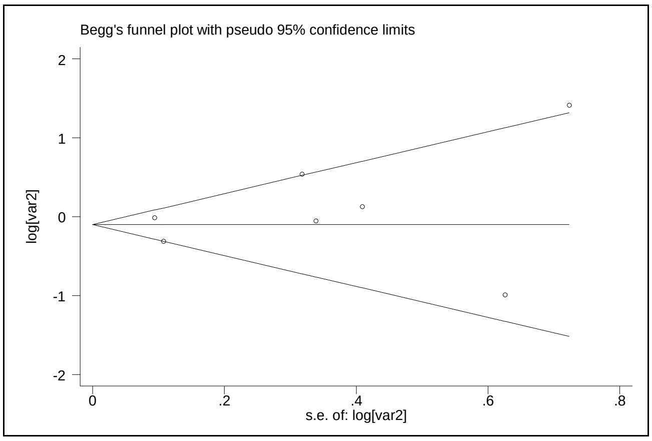

B

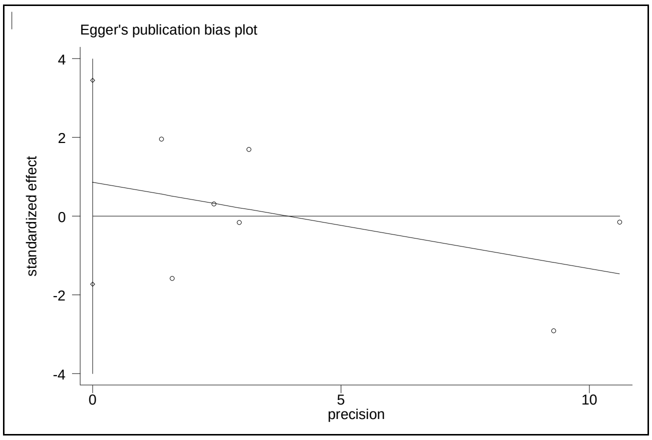

Figure S2. Publication bias test for mRS (0-2) at 3 months. A, Begg's test, z = 0.30 (continuity corrected); $p > |z| = 0.76$ (continuity corrected). B, Egger's test, $p = 0.43$; 95% CI, -1.73–3.45.

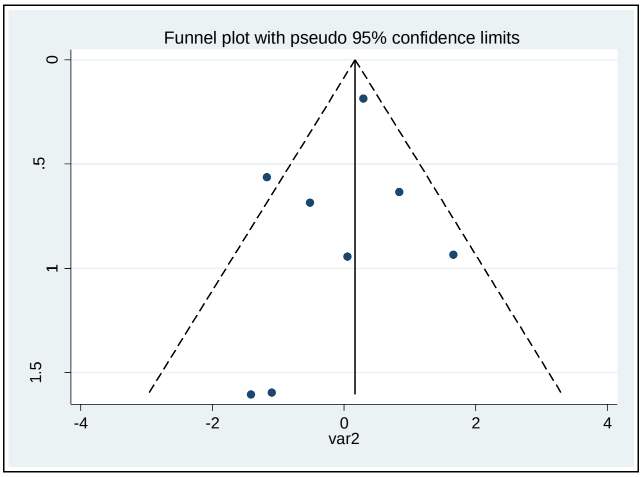

Figure S3. Funnel plot for publication bias test for mortality at 3 months.

A

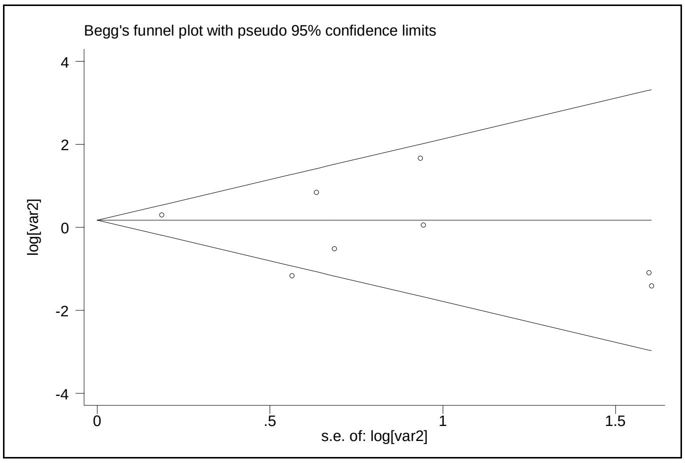

B

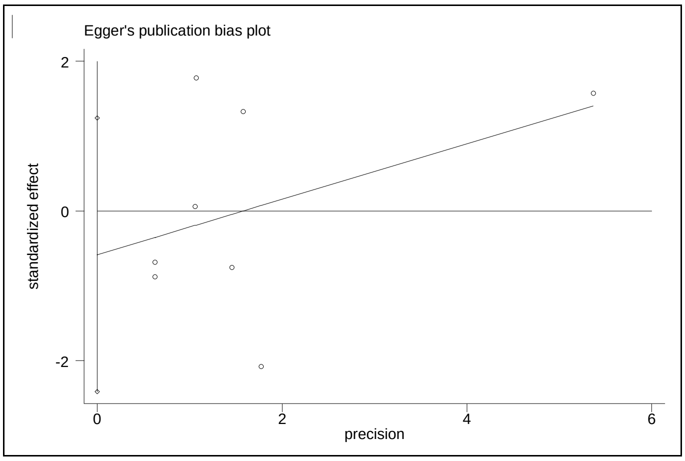

Figure S4. Publication bias test for mortality at 3 months. A, Begg's test, z = 0.37 (continuity corrected); $p > |z| = 0.71$ (continuity corrected). B, Egger's test, $p = 0.46$; 95% CI, -2.41–1.24.
